# Midodrine in patients with ACLF and ascites: When increasing blood pressure is not enough

**DOI:** 10.1097/HC9.0000000000001028

**Published:** 2026-07-27

**Authors:** Enrico Pompili, Martina Salvati, Paolo Caraceni

**Affiliations:** 1 Department of Medical and Surgical Sciences, Alma Mater Studiorum—University of Bologna, Bologna, Italy; 2 Unit of Semeiotics, Liver and Alcohol-Related Diseases, IRCCS Azienda Ospedaliero-Universitaria di Bologna, Bologna, Italy; 3 Department of Internal Medicine, Research Unit of Geriatrics and Internal Medicine, Campus Bio-Medico University of Rome, Rome, Italy

Midodrine is a mild alpha-1 adrenergic agonist commonly used to treat orthostatic and neuro-mediated hypotension in the general population. As an oral agent suitable for both inpatient and outpatient use, midodrine has been proposed as a pharmacological strategy to counteract the marked vasodilation associated with decompensated cirrhosis, particularly after large-volume paracentesis and in patients with refractory ascites or hepatorenal syndrome.^1^ Although several studies and pilot trials have suggested that midodrine may improve systemic hemodynamics and aid ascites control, robust supporting evidence is still lacking.^2^

Acute-on-chronic liver failure (ACLF) remains one of the most challenging syndromes in hepatology. Its pathophysiology is complex and multifactorial, involving hepatic injury, portal hypertension, systemic inflammation, immune dysfunction, circulatory derangement, and extrahepatic organ failures.^3^ Despite increasing recognition of ACLF as a distinct clinical entity, specific disease-modifying treatments remain limited, and management is still largely based on treatment of precipitating events and organ support.^4^ A further obstacle to therapeutic development has been the coexistence of different regional definitions, which identify partially overlapping but clinically distinct populations. This heterogeneity has hampered comparability across studies and complicated trial design. In this context, while we await a harmonized definition of ACLF in the near future, new trials in ACLF populations remain highly valuable.

We therefore read with interest the randomized MIDAS trial by Kulkarni et al,^5^ a double-blind, placebo-controlled study evaluating the addition of oral midodrine to standard medical therapy in 130 patients with ascites and ACLF according to the Asian-Pacific Association for the Study of the Liver (APASL).

The primary endpoint of the MIDAS trial was transplant-free survival at 1 month, while clinically meaningful secondary endpoints were control of ascites and incidence of complications or hospitalizations. Changes in mean arterial pressure (MAP) and plasma renin activity (PRA), as a biomarker of effective arterial blood volume, were also measured.

Unfortunately, the trial was largely negative. Indeed, midodrine did not improve survival or significantly reduce complications and hospitalizations. Moreover, although midodrine significantly increased MAP and was associated with a numerical reduction in PRA that did not reach statistical significance, these hemodynamic changes did not translate into clinical benefit. Finally, midodrine administration appeared safe in this fragile population.

This dissociation between hemodynamic improvement and clinical efficacy is not entirely unexpected. ACLF is a complex systemic syndrome in which the multiple mechanisms interact dynamically and often amplify one another. Within such a multifactorial pathophysiological framework, it may be unrealistic to expect that correction of a single hemodynamic parameter alone can substantially modify the overall clinical course of the syndrome or significantly improve short-term survival.

However, these negative results should not obscure some positive signals regarding the management of ascites. In the whole study population, midodrine treatment was associated with a trend toward better ascites control at 30 days and reduced need for paracenteses, with a significantly lower proportion of patients requiring multiple paracenteses. The subgroup analysis according to baseline MAP is particularly thought-provoking, indicating that patients with low baseline MAP (ie, below 80 mm Hg) appeared to derive a greater short-term benefit in controlling ascites.

Since these analyses were post hoc and the sample size was not adequately powered for such evaluations, the results should be interpreted with caution. Nevertheless, they suggest that midodrine may be more likely to provide clinical benefit when administered to patients who exhibit the specific pathophysiological abnormality targeted by the drug. More broadly, these findings support the concept that vasoconstrictor therapy may be more effective in patients whose clinical phenotype is predominantly characterized by systemic vasodilation and arterial underfilling.

Midodrine dosing and duration of therapy also deserve consideration. To reduce pill burden and facilitate treatment adherence, the authors selected a dose lower than the maximal doses previously used in patients with decompensated cirrhosis. At first glance, this choice may raise the possibility that a more intensive titration strategy could have produced a greater clinical effect. However, midodrine still achieved a meaningful hemodynamic improvement, suggesting that the drug was pharmacologically active in this population and that the administered dose was sufficient to exert its expected circulatory effects. In this context, it cannot be assumed that higher doses would necessarily have translated into improved clinical outcomes, particularly in a complex and multifactorial syndrome such as ACLF. Similarly, midodrine was administered for only 30 days. Therefore, it cannot be excluded that the initial benefits observed on ascites control might become more evident over the longer term, particularly after resolution of the acute phase of ACLF. It is conceivable that, once systemic inflammation and organ dysfunction improve, the hemodynamic effects of midodrine could translate into more sustained clinical benefits in selected patients.

Finally, the co-administration of albumin for ascites management beyond the 3 conventional indications recommended by international guidelines (ie, large-volume paracentesis, spontaneous bacterial peritonitis, and acute kidney injury/hepatorenal syndrome) also deserves consideration. In the MIDAS study, albumin administration was not protocolized, and any conclusion regarding its interaction with midodrine should therefore be interpreted cautiously. Indeed, the non-standardized use of albumin may have diluted or confounded the effect specifically attributable to midodrine, particularly since both interventions target, at least in part, overlapping hemodynamic mechanisms. Furthermore, patients who received albumin appeared to have better ascites control, improved tolerance to diuretic therapy, and a lower incidence of acute kidney injury, findings that are broadly consistent with both controlled trials and real-world experiences evaluating long-term albumin administration for the management of ascites.^6^^–^^9^ However, no significant effect was observed on other complications of cirrhosis or on survival. Interestingly, the cumulative dose of albumin administered was ~95 g per patient over 6 weeks, which is substantially lower and of shorter duration than the regimens used in previous studies reporting broader clinical benefits. Therefore, it is possible that the full therapeutic effects of long-term albumin administration require a more prolonged treatment, potentially extending beyond 2–3 months, and higher cumulative doses than those administered in the present study.

In conclusion, the MIDAS trial does not support the routine use of midodrine to improve survival or prevent complications in patients with APASL-defined ACLF and ascites. However, the study should not be interpreted simply as a negative trial. It confirms that midodrine is generally safe in this setting, demonstrates that the drug can effectively improve systemic hemodynamics, and suggests that selected patients, particularly those with lower baseline mean arterial pressure, may derive more favorable ascites-related benefits. In this respect, the study reinforces the increasingly important concept that therapeutic strategies in patients with complex diseases are unlikely to follow a “one-size-fits-all” approach. The role of midodrine in ACLF, and more broadly in decompensated cirrhosis with ascites, does not lie in widespread empirical use. In the era of precision medicine, future studies using clinically meaningful, yet realistically achievable endpoints should focus on identifying the patient phenotypes most likely to benefit from midodrine, while also optimizing dosing regimens and treatment duration. The ultimate aim is to determine whether an inexpensive, widely available, and apparently safe drug such as midodrine can offer a clinically meaningful benefit in selected subsets of these highly complex and fragile patients.
